# Risk factors for human *Leptospira* seropositivity in South Germany

**DOI:** 10.1186/s40064-016-3483-8

**Published:** 2016-10-18

**Authors:** Stefan O. Brockmann, Lena Ulrich, Isolde Piechotowski, Christiane Wagner-Wiening, Karsten Nöckler, Anne Mayer-Scholl, Martin Eichner

**Affiliations:** 1 Local Public Health Office, Landratsamt Reutlingen, Reutlingen, Germany; 2 Department of Clinical Epidemiology and Applied Biometry, University of Tübingen, Silcherstr. 5, 72076 Tübingen, Germany; 3 Ministry of Health Baden-Württemberg, Stuttgart, Germany; 4 Baden-Württemberg State Health Office Stuttgart, Stuttgart, Germany; 5 Federal Institute for Risk Assessment, Berlin, Germany

## Abstract

We analyzed risk factors for *Leptospira* seropositivity in humans, using data from a population-based cross-sectional zoonosis survey in South Germany (2008/9). Out of 1007 participants 42 (4.2 %) were sero-positive (19/446 men; 23/561 women), indicating that *Leptospira* exposure and sero-conversion is much more frequent than commonly assumed. Relative risks (RR) for seropositivity with exact 95 % confidence intervals (CI; adjusted for specificity and sensitivity of the ELISA test) were calculated for various exposure factors. Contact with pet rats (RR = 13.9 CI [4.8; 25.3]), guinea pigs (3.0[1.1; 7.4]), cattle (3.7[1.3; 9.6]), poultry (3.6[1.3; 8.6]) or livestock (2.3[1.1; 4.9]) as well as occupation as forestry worker (9.2[2.6; 21.4]) were identified as important exposure factors. None of the participants has ever been diagnosed with leptospirosis, yet 45 had experienced symptoms which may have been caused by *Leptospira* infection (12 with scleral icterus, 25 dark urine, 8 liver inflammation, 7 kidney failure). Three times as many participants with prior symptoms were seropositive as participants without symptoms (RR = 3.4[1.3; 8.3]), suggesting that sero-positive patients with severe symptoms may frequently not be diagnosed as leptospirosis cases. Physicians should consider leptospirosis as a differential diagnosis. Currently, the vast majority of symptomatic leptospirosis patients may neither be diagnosed nor reported.

## Background

To date, 20 *Leptospira* species with more than 300 serovars have been described. On the molecular level, *Leptospira* can be divided into nine pathogenic, five intermediate and six environmental non-pathogenic species. Leptospirosis may be the geographically most widespread zoonotic disease worldwide (Levett 2001). Pathogenic *Leptospira* primarily infect wild and domestic animals, which can act as reservoir (Burkhardt et al. 2009). Some serovars have adapted to certain species of mammals in which they only cause mild or no disease (Guerra 2009). Infected hosts excrete *Leptospira* and contaminate water and soil via urine (Burkhardt et al. 2009) where the bacteria can survive for several weeks (Smith and Self 1955). *Leptospira* penetrate into humans through micro-lesions of the skin or through the intact mucosa (Stephan et al. 2000). The incubation period usually takes five to 14 days (Burkhardt et al. 2009). Leptospirosis ranges in severity from a mild, self-limited febrile disease to a fulminant life-threatening illness. The symptoms of leptospirosis are very variable, manifestations range from nonspecific influenza-like symptoms with mild fever, chills, myalgia and headache to kidney and liver failure, associated with high mortality (McGovern et al. 2007). The clinical course of an infection differs depending on the serovar and the patient’s immune status. The most clinically recognizable form of leptospirosis is Weil’s syndrome, a symptom complex which involves kidney failure, jaundice and thrombocytopenia (Inada et al. 1916). Serological diagnosis is the most important means of detecting *Leptospira* infections. Specific antibodies appear in the blood around the fifth day after onset of symptoms; they peak after two or three weeks and can persist for years. The microscopic agglutination test (MAT) (Krauss et al. 1997), still is the gold standard diagnostic method (Burkhardt et al. 2009). The ELISA can detect IgG and IgM and shows more accurate and precise results than MAT (Schlichting et al. 2015). Detection of *Leptospira* DNA is useful for diagnosis prior to sero-conversion (Guerra et al. 2008). From 2001 to 2015, an average of 87 clinical leptospirosis cases were reported annually (range: 46–177) (Robert-Koch-Institut 2015), leading to the assumption that leptospirosis is rare in Germany, yet the underlying incidence of clinical or asymptomatic infections is unknown. Clinical cases frequently were reported in crop workers, farmers, or sewer workers; some water-related recreational activities or having contact to pets or livestock were also reported as risk factors (Jansen et al. 2005). To quantify the influence of potential exposure factors on sero-positivity, we analyzed sero-samples from a randomly selected cross-sectional population samples. The individuals’ responses to exposure questions in a standardized questionnaire were used to determine factors which increase the risk for *Leptospira* infection in humans.

## Methods

We analyzed data from a randomized population-based cross-sectional zoonosis study in Baden-Württemberg, Germany. The study was conducted from April 2008 to December 2009 by the Baden-Württemberg State Health Office. Participation was voluntary; the sample size included 1050 people from nine municipalities (eight counties). The study participants completed a standardized questionnaire asking about possible exposure factors for zoonotic infections such as contact to animals, leisure activities including water sports, occupation, having a domicile near a water body, etc. In order to quantify the number of individuals who had been exposed to *Leptospira* and seroconverted, IgG antibodies were determined from blood samples by the German Consiliary Laboratory (CL) for Leptospirosis using an IgG in-house ELISA (Schlichting et al. 2015). This study has been approved by the ethics committee of the State Medical Chamber of Baden-Württemberg and has been performed in accordance with the ethical standards (Ref Nr. 2008-024-f). Written informed consent was obtained from each participant. The IgG serum values were regarded “positive” (>98 OD %), “questionably positive” (86–98 OD %) or “negative” (<86 OD %) as defined by the CL. Statistical analyses were done with JMP 11 (SAS-Institute 2014) and R 3.0.2 (The-R-Foundation-for-Statistical-Computing 2014). The Relative Risk (RR) expresses the factor by which the probability of *Leptospira* IgG positivity differs between “exposed” and “non-exposed” individuals. As the questionnaire frequently allowed more than two answers to exposure questions (e.g. contacts occurred “never”, “rarely”, “occasionally”, “frequently”), we had to group answers before calculating RRs: “frequent” and “occasional” were compared to “rare” and “never”. Exact 95 % confidence intervals (CI) of the RR were calculated with a specific R package, because numbers were too small to employ the frequently used calculation based on normal approximation (Ludwig-Mayerhofer 2014). Schlichting et al. report that the sensitivity (se) and specificity (sp) of the ELISA test for sub-clinical infections are se = 85.7 % and sp = 99.1 %, respectively (Schlichting et al. 2015). This means that test-positive (pos) and test-negative (neg) observations do not fully reflect the true prevalence (p) of sero-positivity in any one of the groups. Considering sensitivity and specificity, the numbers of positive and negative observations are given by ${\text{pos}} = {\text{n}}*{\text{p}}*{\text{se}} + {\text{n}}*\left( { 1 - {\text{p}}} \right)*\left( { 1 - {\text{sp}}} \right){\text{ and n}} = {\text{neg}} + {\text{pos}}.$ Solving these equations for p, we obtain the expected true prevalence

$$p = \left\{ \begin{aligned} \,\,\,\,\,\,\,0\,\,\,\,\,\,\,\,\,\,\,\,\,\,\,\,\,\,\,\,\,\,\,\,\,\,\,if\,\,pos/n \le 1 - sp \hfill \\ \,\,\,\,\,\,\,\,1\,\,\,\,\,\,\,\,\,\,\,\,\,\,\,\,\,\,\,\,\,\,\,\,\,\,\,if\,\,pos/n \ge se \hfill \\ \frac{{\frac{pos}{n} - (1 - sp)}}{se - (1 - sp)}\,\,\,\,\,\,\,\,otherwise \hfill \\ \end{aligned} \right.$$

(assuming se > 50 % and sp > 50 % as is the case here).

We corrected the number of positive findings accordingly and re-calculated the corresponding RRs (Tables 1, 2, 3, 4). As exact CIs for these RRs could not be calculated based on the decimal (non-integer) number of corrected positive findings, we rounded each number of positive findings to the next lower and the next higher number, and calculated CIs for each one of the resulting four combinations. Finally, we reported the lowest and highest obtained value of these four CIs (resulting in conservative CIs).

**Table 1:** Direct contact with animals

Comparison of exposure groups “frequent” or “occasional” vs. “rare” or “never”	Proportion of *Leptospira* IgG positive subjects			Corrected		
	Exposed	Non-exposed	RR [95 % CI]	Exposed	Non-exposed	RR [95 % CI]
*Rat (pet)*	*41.7* *% (5/12)*	*3.8* *% (37/964)*	*10.9 [4.6; 20.1]*	*48.1* *% (5.8/12)*	*3.5* *% (33.4/964)*	*13.9 [4.8; 25.3]*
*Cattle (livestock)*	*12.5* *% (4/32)*	*4.0* *% (38/947)*	*3.1 [1.2; 7.5]*	*13.7* *% (4.4/32)*	*3.7* *% (34.8/947)*	*3.7 [1.3; 9.6]*
*Poultry (livestock)*	*11.9* *% (5/42)*	*4.0* *% (37/931)*	*3.0 [1.2; 6.8]*	*13.0* *% (5.5/42)*	*3.6* *% (33.8/931)*	*3.6 [1.3; 8.6]*
Mouse (pet)	11.5 % (3/26)	4.1 % (39/950)	2.8 [0.9; 7.4]	12.5 % (3.3/26)	3.8 % (35.9/950)	3.3 [0.9; 9.8]
*Guinea pig (pet)*	10.2 % (5/49)	4.0 % (37/927)	2.6 [1.0; 5.8]	*11.0* *% (5.4/49)*	*3.6* *% (33.8/927)*	*3.0 [1.1; 7.4]*
*Any livestock animal*	7.5 % (10/133)	3.7 % (32/855)	2.0 [1.0; 3.9]	*7.8* *% (10.4/133)*	*3.4* *% (28.7/855)*	*2.3 [1.1; 4.9]*
Cat (pet)	5.7 % (22/386)	3.3 % (20/602)	1.7 [0.9; 3.1]	5.7 % (21.8/386)	2.9 % (17.2/602)	2.0 [1.0; 3.8]
Any pet	4.9 % (31/636)	3.0 % (11/365)	1.6 [0.8; 3.3]	4.7 % (29.8/636)	2.5 % (9.1/365)	1.9 [0.8; 4.0]
Hare (pet)	6.3 % (5/80)	4.1 % (37/892)	1.5 [0.6; 3.5]	6.3 % (5.0/80)	3.8 % (34.2/892)	1.6 [0.7; 4.4]
Dog (pet)	5.2 % (19/366)	3.7 % (23/623)	1.4 [0.8; 2.6]	5.1 % (18.5/366)	3.3 % (20.5/623)	1.5 [0.8; 3.0]
Bird (pet)	6.0 % (4/67)	4.2 % (38/912)	1.4 [0.5; 3.6]	6.0 % (4.0/67)	3.9 % (35.1/912)	1.6 [0.6; 4.0]
Horse (livestock)	4.9 % (4/81)	4.2 % (38/897)	1.2 [0.4; 3.0]	4.8 % (3.9/81)	3.9 % (35.3/897)	1.2 [0.3; 3.3]
Goat (livestock)	4.2 % (1/24)	4.2 % (40/952)	1.0 [0.1; 5.0]	3.9 % (0.9/24)	3.9 % (37.1/952)	1.0 [0.0; 5.3]
Rabbit (pet)	3.7 % (4/109)	4.4 % (38/868)	0.8 [0.3; 2.2]	3.3 % (3.6/109)	4.1 % (35.6/868)	0.8 [0.2; 2.4]
Hamster (pet)	2.9 % (1/35)	4.4 % (41/942)	0.7 [0.0; 3.4]	2.3 % (0.8/35)	4.1 % (38.4/942)	0.6 [0.0; 3.7]
Sheep (livestock)	0.0 % (0/23)	4.4 % (42/954)	0.0 [0.0; 3.3]	0.0 % (0/23)	4.1 % (39.4/954)	0.0 [0.0; 3.4]
Pig (livestock)	0.0 % (0/9)	4.3 % (42/967)	0.0 [0.0; 7.3]	0.0 % (0/9)	4.1 % (39.3/967)	0.0 [0.0; 7.7]

Italic values show risk factors whose 95 % CI do not contain the value 1, i.e. they are statistically significant on the 5 % level

**Table 2:** Occupation

Comparison of exposure groups “yes” vs. “no”	Proportion of *Leptospira* IgG positive subjects			Corrected		
	Exposed	Non-exposed	RR [95 % CI]	Exposed	Non-exposed	RR [95 % CI]
Gardener	3.0 % (1/33)	1.1 % (10/919)	2.8 [0.3; 15.8]	2.5 % (0.8/33)	0.2 % (2.0/919)	11.3 [0.0; 119.9]
*Forestry worker*	*30.0* *% (3/10)*	*4.0* *% (38/939)*	*7.4 [2.4; 6.3]*	*34.3* *% (3.4/10)*	*3.7* *% (34.8/939)*	*9.2 [2.6; 21.4]*
Hunter	12.5 % (1/8)	4.2 % (40/944)	3.0 [0.2; 1.7]	13.7 % (1.1/8)	3.9 % (37.2/944)	3.5 [0.2; 16.6]
Farmer	8.3 % (3/36)	4.1 % (38/920)	2.0 [0.6; 5.6]	8.8 % (3.2/36)	3.8 % (35.0/920)	2.3 [0.6; 7.1]
Seeing rodents at work	7.7 % (3/39)	4.1 % (39/956)	1.9 [0.6; 5.2]	8.0 % (3.1/39)	3.7 % (35.8/956)	2.1 [0.6; 6.8]
Any outdoor occupation	5.1 % (7/137)	4.1 % (35/855)	1.2 [0.6; 2.7]	5.0 % (6.8/137)	3.8 % (32.2/855)	1.3 [0.5; 3.0]
Other outdoor occupation not mentioned in this table	2.7 % (2/74)	4.5 % (39/874)	0.6 [0.1; 2.1]	2.1 % (1.6/74)	4.2 % (36.7/874)	0.5 [0.0; 2.3]
Veterinarian	0.0 % (0/1)	4.3 % (41/947)	0.0 [0.0; 22.5]	0.0 % (0/1)	4.0 % (38.3/947)	0.0 [0.0; 24.2]
Waste worker	0.0 % (0/12)	4.4 % (41/938)	0.0 [0.0; 5.7]	0.0 % (0/12)	4.1 % (38.4/938)	0.0 [0.0; 6.1]
Shepherd	0.0 % (0/3)	4.3 % (41/944)	0.0 [0.0; 14.5]	0.0 % (0/3)	4.1 % (38.3/944)	0.0 [0.0; 15.5]

Italic values show risk factors whose 95 % CI do not contain the value 1, i.e. they are statistically significant on the 5 % level

**Table 3:** Recreational activities

Comparison of exposure groups “frequent” or “occasional” vs. “rare” or “never”	Proportion of *Leptospira* IgG positive subjects			Corrected		
	Exposed	Non-exposed	RR [95 % CI]	Exposed	Non-exposed	RR [95 % CI]
Surfing/kiting	33.3 % (1/3)	4.1 % (41/989)	8.0 [0.5; 1.2]	38.2 % (1.1/3)	3.8 % (37.9/989)	10.0 [0.6; 29.6]
Sailing	14.3 % (1/7)	4.2 % (41/985)	3.4 [0.2; 3.1]	15.8 % (1.1/7)	3.8 %(37.9/985)	4.1 [0.2; 19.4]
Diving	11.1 % (1/9)	4.2 % (41/983)	2.7 [0.2; 1.0]	12.0 % (1.1/9)	3.9 % (37.9/983)	3.1 [0.2; 15.9]
Any watersport	8.9 % (5/56)	3.9 % (37/944)	2.3 [0.9; 5.3]	9.5 % (5.3/56)	3.6 % (33.6/944)	2.7 [1.0; 6.7]
Canoeing, kayaking, rowing	6.3 % (1/16)	4.2 % (41/976)	1.5 [0.1; 7.0]	6.3 % (1.0/16)	3.9 % (38.0/976)	1.6 [0.1; 10.1]
Bathing in inland waters	5.6 % (9/160)	3.9 % (33/842)	1.4 [0.7; 2.9]	5.6 % (8.9/160)	3.6 % (30.0/842)	1.6 [0.7; 3.3]
Traveling to tropics	5.3 % (9/171)	4.0 % (33/830)	1.3 [0.6; 2.7]	5.1 % (8.8/171)	3.6 % (30.1/830)	1.4 [0.6; 3.0]
Other watersports not mentioned in this table	3.6 % (1/28)	4.4 % (39/886)	0.8 [0.1; 4.1]	3.2 % (0.9/28)	4.1 % (36.6/886)	0.8 [0.0; 4.7]
Traveling to Mediterranean	3.8 % (27/713)	5.2 % (15/288)	0.7 [0.4; 1.4]	3.4 % (24.3/713)	5.1 % (14.6/288)	0.7 [0.3; 1.4]
Rod fishing	0.0 % (0/10)	4.3 % (42/984)	0.0 [0.0; 6.9]	0.0 % (0/10)	4.0 % (39.1/984)	0.0 [0.0; 7.4]

**Table 4:** Residence-related exposure

Comparison of exposure groups “yes” vs. “no”	Proportion of *Leptospira* IgG positive subjects			Corrected		
	Exposed	Non-exposed	RR [95 % CI]	Exposed	Non-exposed	RR [95 % CI]
Home close to forest (<100 m)	6.1 % (6/98)	4.0 % (36/900)	1.5 [0.7; 3.4]	6.2 % (6.0/98)	3.7 % (32.9/900)	1.7 [0.7; 4.3]
Seeing rodents around home	5.7 % (4/70)	4.1 % (38/932)	1.4 [0.5; 3.5]	5.7 % (4.0/70)	3.7 % (34.9/932)	1.5 [0.4; 4.0]
Stockpiling firewood close to home	4.7 % (27/574)	3.5 % (14/397)	1.3 [0.7; 2.5]	4.5 % (25.7/574)	3.1 % (12.3/397)	1.4 [0.7; 3.0]
Home close to inland water (<100 m)	4.0 % (24/607)	4.6 % (18/391)	0.9 [0.5; 1.6]	3.6 % (21.9/607)	4.4 % (17.1/391)	0.8 [0.4; 1.6]
Home with garden pond	3.0 % (7/231)	4.4 % (27/618)	0.7 [0.3; 1.5]	2.5 % (5.8/231)	4.1 % (25.3/618)	0.6 [0.2; 1.5]
Home with garden	3.6 % (28/771)	6.1 % (14/231)	0.6 [0.3; 1.1]	3.2 % (24.8/771)	6.1 % (14.1/231)	0.5 [0.3; 1.0]

## Results

Out of 1050 participants, 43 had questionably positive IgG serum levels and, therefore, had to be excluded. 42 of 1007 subjects (4.2 %) had positive *Leptospira* IgG serum levels. The age of these 1007 participants ranged from 17 to 66 years. Among men, 19/446 (4.3 %) were positive; among women, it were 23/561 (4.1 %). We calculated RRs for seropositivity for all questions in the questionnaire (Tables 1, 2, 3, 4). Direct contact to animals in general or occupation as forestry worker led to the highest RRs. Particularly, contact with pet rats, guinea pigs, cattle, poultry or livestock in general yielded high RR values with CI which excluded the value 1 (Table 1). Among the occupations, only forestry workers yielded a high RR whose CI excluded the value 1 (Table 2) whereas the RRs for other occupations were comparatively low with large CIs. Neither bathing in inland waters nor any of the water sports conclusively led to elevated RRs (Table 3). None of the residence-related exposure factors showed significantly increased RR values (Table 4) and none of the participants had been diagnosed with leptospirosis before. A total of 45 participants had experienced symptoms without specific diagnosis which are common for leptospirosis (12 participants had had scleral icterus; 25 dark urine; 8 liver inflammation; 7 kidney failure) within the last five years. Among them, 5 were seropositive (3 men, 2 women); contrasting this with 37 seropositive findings in 957 participants without any of these symptoms, we obtained a RR of 2.9 (with 95 % CI [1.2; 6.5]), i.e. three times as many participants with prior symptoms were seropositive as participants without symptoms. After correcting for sensitivity and specificity of the test, we obtained a RR = 3.4 [1.3; 8.3]. None of the 5 seropositive participants with symptoms reported a diagnosis of a zoonotic disease like Q fever, hantavirus or Hepatitis E which would explain these symptoms.

## Discussion

**Fig. 1:**
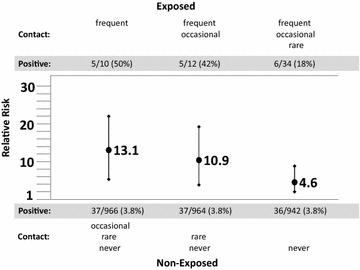
Uncorrected relative Risks (RR; points with associated values) and their exact 95 % confidence intervals (*vertical lines*) for *Leptospira* IgG sero-positivity (“exposed” vs. “non-exposed”), using different exposure groupings of “rat contact”. The data were collected in the course of the population-based cross-sectional zoonosis study from April 2008 to December 2009 by the Baden-Württemberg State Health Office Baden-Württemberg, Germany. The *horizontal line* marks RR = 1. The allocation of the subjects to the groups of “exposed” and “non-exposed” is displayed above and below the graph

Our findings confirm the importance of contact with animals, specifically with pet rats, as exposure factor to *Leptospira* infection. Five of 12 persons (42 %) who reported frequent or occasional rat contact were IgG positive, whereas 37 of 964 persons (3.8 %) who rarely or never had rat contact were IgG positive, resulting in a RR of 10.9. Grouping the answers differently had a major impact on the results: regarding only “frequent contact” as exposure increased the RR from 10.9 to 13.1 (associated with a broader confidence interval as the number of exposed individuals became smaller); regarding only individuals who never had any contact with pet rats as non-exposed reduced it to 4.6 with a narrower confidence interval whose lower limit was 2.0 (Fig. 1).

Apart from contact with pet rats, the contact with guinea pigs, cattle, poultry or livestock in general, as well as occupation as forestry worker led to RRs whose confidence intervals did not contain 1.0 (Tables 1, 2, 3, 4), indicating that these exposures also increased exposure to *Leptospira* spp., although care should be taken when interpreting these results. By definition, only 95 % of the 90 confidence intervals presented in this paper contain the true RR. Thus, we expect that 4.5 confidence intervals do not contain the true RR (i.e. it could be beyond the lower or the upper limit of the reported interval). After Bonferroni-Holm correction, only rat contacts remain a statistically significant exposure factor. Moreover, as each participant of the study was supposed to answer every question, some of the associations between seropositivity and exposure found in this study may simply be due to confounding of different exposure factors. As there were only 42 seropositive individuals, any attempt of disentangling such confounders or of making a multivariate analysis was futile. We also investigated some well-known literature-confirmed risk factors like water sports, trips abroad, being a farmer, or pig farmer (Forbes et al. 2012; WHO 2003). Due to the very low number of exposed subjects in some of these categories, statistical power is low, which leads to non-significant results unless the RR is huge.

Interestingly, participants who reported having had typical leptospirosis symptoms without diagnosis during the last five years, were three times as likely to be seropositive. Other seropositive participants may have had similar symptoms a longer time ago. If, indeed, the 5 observed symptomatic sero-positive participants were undetected leptospirosis patients, the estimated prevalence of unreported cases in our collective would be 0.5 % (or even higher), indicating that roughly 10 % of seropositive participants may have experienced leptospirosis. The results of our study suggest that *Leptospira* exposure and sero-conversion in Baden-Wuerttemberg may be more widespread than previously assumed. In contrast, the reported annual disease incidence is only 0.0953 per 100,000 (Robert-Koch-Institut 2015). Applying this figure to our population of 1050 participants with an average age of 45 years, we expect that during the lifetime of these participants, 0.045 cases would have been reported. This would be in gross contrast to the assumed 5 leptospirosis cases, indicating a dark figure of 99.1 % which are neither diagnosed nor treated for leptospirosis. The overall number of IgG positive subjects in our study was 42 or (after correction for sensitivity and specificity) 40.5, indicating that even 99.9 % of (asymptomatic or symptomatic) infections remain unknown. Particular serotypes which cause mild or asymptomatic infections may occur more frequently in Baden-Wuerttemberg than currently assumed. It has been reported in the literature that leptospirosis frequently is under- or misdiagnosed in Germany (Brockmann et al. 2010; Abela-Ridder et al. 2010). Although there were no relevant differences in seropositivity between male and female subjects in our cross-sectional study, 3/446 (0.67 %) of men and 2/561 (0.36 %) of women were seropositive and reported typical leptospirosis symptoms, confirming the gender difference of about 2:1 in notified leptospirosis cases in Germany (Robert-Koch-Institut 2015).

## Conclusions

Our study confirms that contact with pet rats plays an important role in the acquisition of human leptospirosis. Specifically, rat owners have to be aware of the exposure risk to pathogenic leptospires and should not let house rats stroll around outside where they might encounter wild rats or their excrements and, thus, get infected with *Leptospira*. Additional sources of infection (e.g. contact with other pets or livestock) also exist; infection with *Leptospira* is much more frequent than commonly assumed. Furthermore, most sero-positive patients with severe symptoms are not diagnosed as leptospirosis cases. Physicians, therefore, should consider leptospirosis as a differential diagnosis. Currently, the vast majority of symptomatic leptospirosis patients may neither be diagnosed nor reported.
